# Electrically Enhanced Transition Metal Dichalcogenides as Charge Transport Layers in Metallophthalocyanine-Based Solar Cells

**DOI:** 10.3389/fchem.2020.612418

**Published:** 2020-12-04

**Authors:** Lebogang Manamela, Juvet N. Fru, Pannan I. Kyesmen, Mmantsae Diale, Nolwazi Nombona

**Affiliations:** ^1^Department of Chemistry, University of Pretoria, Pretoria, South Africa; ^2^Department of Physics, University of Pretoria, Pretoria, South Africa

**Keywords:** phthalocyanines, charge transporting layer, exfoliation, N-type, TMDS

## Abstract

Transitional metal dichalcogenides (TMDs), such as molybdenum disulfide (MoS_2_) have found application in photovoltaic cells as a charge transporting layer due to their high carrier mobility, chemical stability, and flexibility. In this research, a photovoltaic device was fabricated consisting of copper phthalocyanine (CuPc) as the active layer, exfoliated and Au-doped MoS_2_, which are n-type and p-type as electron and hole transport layers, respectively. XRD studies showed prominent peaks at (002) and other weak reflections at (100), (103), (006), and (105) planes corresponding to those of bulky MoS_2_. The only maintained reflection at (002) was weakened for the exfoliated MoS_2_ compared to the bulk, which confirmed that the material was highly exfoliated. Additional peaks at (111) and (200) planes were observed for the Au doped MoS_2_. The interlayer spacing (d_002_) was calculated to be 0.62 nm for the trigonal prismatic MoS_2_ with the space group *P*6*m*2. Raman spectroscopy showed that the ${\text{E}}_{2}^{1}$g (393 cm^−1^) and A_1g_ (409 cm^−1^) peaks for exfoliated MoS_2_ are closer to each other compared to their bulk counterparts (378 and 408 cm^−1^, respectively) hence confirming exfoliation. Raman spectroscopy also confirmed doping of MoS_2_ by Au as the Au-S peak was observed at 320 cm^−1^. Exfoliation was further confirmed by SEM as when moving from bulky to exfoliated MoS_2_, a single nanosheet was observed. Doping was further proven by EDS, which detected Au in the sample suggesting the yield of a p-type Au-MoS_2_. The fabricated device had the architecture: Glass/FTO/Au-MoS_2_/CuPc/MoS_2_/Au. A quadratic relationship between *I-V* was observed suggesting little rectification from the device. Illuminated *I-V* characterization verified that the device was sensitive and absorbed visible light. Upon illumination, the device was able to absorb photons to create electron-hole pairs and it was evident that semipermeable junctions were formed between Au-MoS_2_/CuPc and CuPc/MoS_2_ as holes and electrons were extracted and separated at respective junctions generating current from light. This study indicates that the exfoliated and Au-MoS_2_ could be employed as an electron transporting layer (ETL) and hole transporting layer (HTL), respectively in fabricating photovoltaic devices.

## Introduction

Climate change and global warming occur mainly due to the release of CO_2_ and other greenhouse gases into the atmosphere (Hewage et al., 2019). These greenhouse gases trap heat on the earth's surface and atmosphere increasing the average temperature of the globe. The release of these harmful gases is mainly due to the burning of fossil fuels for energy generation. This suggests the need for the implementation of better technologies to mitigate this issue of climate change (Perera, 2018).

Solar cells are devices that have been developed to capture photons from the sun to produce electricity (Wang et al., 2014; Yu and Sivula, 2017). The major advantage of using solar cells is that they are small devices that can be used anywhere unlike current electrical technologies. Adding on to their advantages, the cost of the solar cell can be reduced by making use of cheap materials and they only require the abundant and free solar energy as a source (Shah and Wallace, 2004; Guo et al., 2008). A typical solar cell consists of an active layer, an electron transporting layer (ETL) (most often an n-type semiconductor), and a hole transporting layer (HTL) (most often a p-type semiconductor) sandwiched between two electrodes (Markvart and Castaner, 2018). The active layer absorbs photons from the sunlight and generate an electron-hole pair. The electron transporting layer acts as a semipermeable membrane which only extracts and separates the electrons from the holes (Markvart and Castaner, 2018). The electron is then taken to the cathode and through an external circuit generating electricity, and finally gets to the anode where it recombines with the hole. The hole would, at the same time, have been extracted by the hole transporting layer, separated from the electron, and ultimately taken to the anode for recombination with the electron that has completed the circuit (Ragoussia and Torres, 2015; Marinova et al., 2016). An efficient solar cell is designed such that the time the charges take to reach their respective membranes (p and n-type semiconductors) is smaller than the lifetime of the electron-hole pair (Shieh et al., 2010; Zhu et al., 2018). However, the abovementioned mechanism does not occur as smoothly. Some solar cells experience frequent electron-hole recombination as their semiconducting materials have low carrier charge mobility. Some solar cells have materials that have low thermal and chemical stability resulting in a reduced lifetime of the solar cell (Dharmadasa et al., 2019).

Solar cells that are currently being extensively studied are nanocrystal based, polymer, perovskite and dye-sensitized solar cells (Luceño-Sánchez et al., 2019). The advantages of these solar cells are their low cost of production, flexibility, lightness, and their ever-improving efficiencies (Yan and Saunders, 2014). Generally, these solar cells have low cost of fabrication as compared with widely used solar cells (silicon-based). However, their efficiencies are relatively low. Perovskite solar cells have efficiencies comparable to silicon solar cells (Ke and Kanatzidis, 2019). They are however disadvantageous as they suffer from chemical and thermal stability (Dharmadasa et al., 2019). This study will utilize the metallophthalocyanines (MPc), CuPc, as a photoactive layer. This is due to its intense light absorption in the visible to near-infrared and they have high chemical and thermal stability compared to the widely used perovskite and polymer photon absorbers. Transition metal dichalcogenides (TMDs) will be employed in this study as charge carrier agents.

TMDs are semiconducting materials with the molecular formula MX_2_, were M is a transition metal (Mo, W, Sn, etc.) and X a chalcogen (S, Se, Te) (Brent et al., 2017). For each TMD, the layer of M atoms is sandwiched between two layers of X atoms and these atoms are held together by covalent bonds. TMDs naturally occur as bulk materials which simply means that many layers of MX_2_ are held together by weak van der Waals forces. In this bulk form, these materials have a bandgap of ~1.2 eV (Choi et al., 2017). When bulky, TMDs can be converted to mono- or few-layers by separating the nanosheets, consequently changing the bandgap to ~1.8 eV (Yun et al., 2012). TMDs have interesting properties, such as high surface area, catalytic active sites and the bandgap can be manipulated (Li and Zhu, 2015). TMDs have high charge carrier mobility (Ke and Kanatzidis, 2019; Zhou and Zhao, 2019), which means that they are able, under an electric field, to move electrons and holes quickly through their surface (Li and Zhu, 2015). This implies that the electrons and the holes will not recombine quickly after electron-hole pair generation resulting in an improved performance of the solar cell. In addition, 2D MoS_2_ materials are advantageous over their bulk, 0D and 1D counterparts because of their excellent electronic, charge storage and catalytic properties including quantum confinement, high absorption coefficient, high surface-to-volume ratio and tunable bandgap (Nawz et al., 2020). These properties have led to TMDs being used in a variety of applications, such as solar cells, lubricants, transistors, and hydrogen evolution catalysis (Mandyam et al., 2020). MoS_2_ is intrinsically n-type and is mostly used as an ETL. However, it can undergo p-type doping with gold (Au) to fit its purpose as the HTL.

The CuPc molecule contains 18-π electrons and the delocalization of these electrons renders it useful for various applications, such as solar cell and other opto-electronic devices. CuPc is chemically and thermally stable (Li et al., 2017). This material is characterized by an intense light absorption in the visible to near infrared making it a good candidate as a photoactive layer in solar cell devices. CuPc, to the best of our knowledge, has not been formerly investigated in photovoltaics as the sole photoactive layer. This material has been applied as an active layer together with a polymer or an organic molecule to form an active layer blend. Kim et al. (2016) fabricated several MPc-based solar cells. The photoactive layers of these solar cells were a blend of MPc and 3,4,9,10-perylenetetracarboxylic bisbenzimidazole (PTCBI) (MPc:PTCBI). The PdPc based solar cell showed the highest efficiency as it reached a PCE of 1.3%. An explanation provided for this result was that PdPc had the longest excitonic diffusion as compared to the other MPc's (ZnPc and CuPc). The results for PdPc-based solar cells showed drastic enhancement of performance when PTCBI was substituted by C_60_ fullerenes. The PCE nearly doubled, as it was reported to be 2.2%. Ali et al. (2020) report the fabrication of an organic solar sell with the active layer being CuPc/C_60_. Derouiche et al. (2010) fabricated a solar cell with the active layer consisting of CuPc:PCBM. For the first time, the use of CuPc individually as the active layer will be reported. This type of solar cell will be cost-effective in comparison to traditional polymer based solar cells that are more complex in their fabrication.

TMD nanosheets have been used in several electronic applications, such as solar cells and field effect transistors. Li et al. (2015) fabricated an ultrathin p-n junction using p-type and n-type MoS_2_. The p-type MoS_2_ was obtained from doping MoS_2_ with gold. The fabricated p-n junction device showed solar to electricity conversion for MoS_2_ with thicknesses that are <8 nm. The film thickness that gave the most efficiency was found to be 3 nm. It was further concluded that ultrathin films were desired since they were flexible, light and transparent, an advantage over the most widely used silicon semiconductor. Ye et al. (2015) fabricated a p-n bilayer photodetector using doped and undoped MoS_2_ (n- and p-type, respectively). They learned that the device has the potential to separate photogenerated charges and observed an improvement in response and sensitivity to the visible light. As such, the device exhibited a desired performance under illumination. Gu et al. (2013) used ultrathin MoS_2_ nanosheets as hole transporting layers in an organic solar cell. The fabricated organic solar cell showed an improved power conversion efficiency (PCE) when compared to the device that used traditional MoO_3_ as the hole transporting layer. Singh et al. (2019) fabricated a perovskite solar cell and used MoS_2_ as the ETL. The solar cell demonstrated a PCE of 13.1% which is comparable to PCE's obtained when “state-of-the-art” compact TiO_2_ and SnO_2_ are used as ETL. This shows that p- and n-type MoS_2_ nanosheets improve charge transport, response and sensitivity of the device to visible light, implying that these materials can add value in photovoltaic devices. The p- and the n-type MoS_2_ have been used in the same p-n junction device and they have also been used as either the ETL or HTL in a single solar cell device, as earlier stated. However, to the best of our knowledge, these materials have never been used together in one solar cell as the HTL and the ETL, respectively. Also, MoS_2_ and CuPc have been used together previous in several applications including FET and sensing. However, to the best of our knowledge, there is no solar cell that consist of both CuPc and MoS_2_.

**Figure 1:**
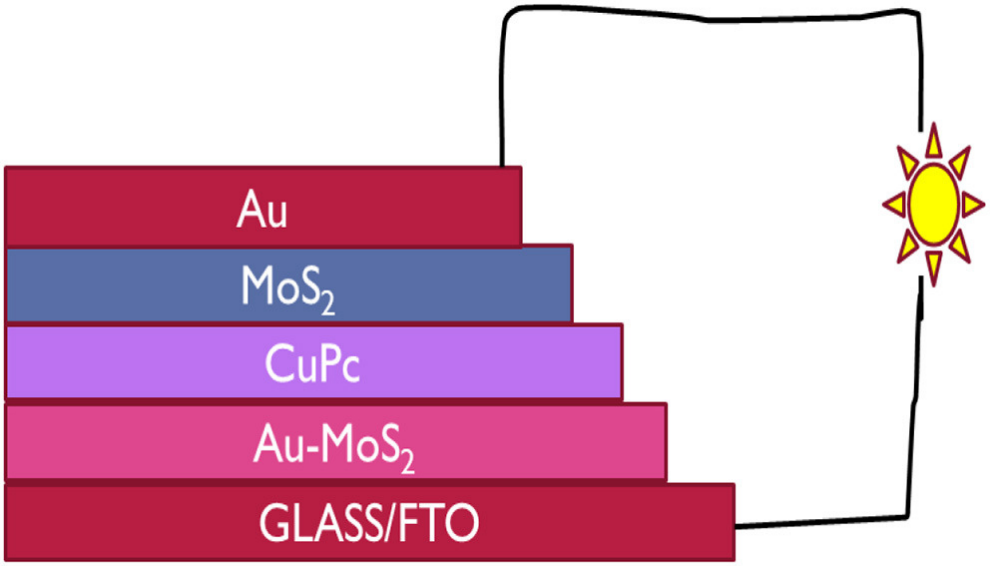
The architecture of the device fabricated in this study with CuPc as the active layer, MoS_2_ and Au-MoS_2_ the ETL and HTL, respectively sandwitched between the two electrodes, FTO and Au contact.

In this study, for the first time, CuPc and MoS_2_ were used in a solar cell. n- and p-type MoS_2_ were used together in a solar cell as an ETL and HTL, respectively and CuPc was used as a photoactive layer. A cost effective device with the architecture Glass/FTO/Au-MoS_2_/CuPc/MoS_2_/Au was fabricated. Au-MoS_2_ was deposited onto the FTO/Glass by spin coating followed by annealing. The next step was the physical vapor deposition which was used to deposit CuPc. This was followed by the deposition of MoS_2_ by spin coating and lastly, the gold contact by physical vapor deposition. Figure 1 illustrates how the device is structured. Under illumination, the device produced photogenerated electron-hole pairs and it was evident that semipermeable junctions were formed between Au-MoS_2_/CuPc and CuPc/MoS_2_ as holes and electrons were extracted and separated at respective junctions generating current from light. This study showed that both exfoliated and doped MoS_2_ could be employed as ETL and HTL, respectively, when fabricating photovoltaic devices.

## Methods and Materials

Synthesis of MoS_2_ nanosheets was carried out in an ultrasonic bath from Labotech (using model 704). Spin coating was carried out using a Laurell technology spin coater model WS-650MZ-23NPPB. Samples were centrifuged using a thermo scientific Labofuge 700 centrifuge.

Acetone, dimethylformamide (DMF), isopropyl alcohol and N-methyl-2-pyrrolidone (NMP) were all purchased from Radchem (Pty) LTD. Copper phthalocyanine (CuPc), fluorine-doped tin oxide (FTO), gold (III) chloride (AuCl_3_), nickel phthalocyanine (NiPc) were purchased from Sigma-Aldrich. Molybdenum disulphide (MoS_2_) was purchased from Protea laboratory solution (Pty) LTD.

### Synthesis of MoS_2_ Nanosheets

MoS_2_ nanosheets were synthesized following a previously reported procedure (Wang et al., 2014). The liquid-based exfoliation method was followed to obtain a large quantity of MoS_2_. Briefly, 0.5 g of MoS_2_ powder was added into 10 mL N-methyl-2-pyrrolidone (NMP) which was the exfoliating agent. The concentration of the resulting mixture was 5 mg·mL^−1^. The mixture was then sonicated for 6 h at 5°C. The mixture was left to settle for 72 h. The upper three-quarters of the mixture was decanted into a centrifuge tube and centrifuged at 3,000 rpm for 30 min and the supernatant was collected and deposited as a thin film on a glass substrate for characterization.

### Doping of MoS_2_ With Gold

Doping of the MoS_2_ by gold was done following a procedure previously reported (Ubani et al., 2016). In summary, 20 mM of aqueous gold solution was prepared by dissolving 0.3 g of AuCl_3_ in distilled water. Ten microliters of the gold solution was spin-coated on TMD coated glass substrate, at 2,500 rpm for 1 min. The resulting sample was annealed at 100°C for 10 min to obtain the Au doped TMDs.

### Solar Cell Fabrication

FTO-coated glass substrates were cleaned thoroughly by sonication in 2-propanol, then in acetone, and lastly in distilled water for 10 min in each solvent. Subsequently, a MoS_2_ dispersed in an organic solvent was spin-coated on FTO at 1,500 rpm. Thereafter, aqueous AuCl_3_ (20 mM) was spin-coated on top of the MoS_2_ layer. The FTO substrate was annealed at 100°C for 10 min. The sample was placed in a physical vapor deposition bell jar where a layer of a CuPc was deposited. The thickness of the MPc film was maintained at 200 nm. Another TMD layer was deposited by spin coating on top of the MPc film. The resulting sample was annealed at 210°C for 60 min. A gold contact of thickness 100 nm was deposited on the substrate using physical vapor deposition.

### Characterization

X-ray diffraction (XRD) analysis was conducted using a Bruker D2 PHASER-e diffractometer using Cu-Kα radiation (0.15418 nm) to identify the phase orientation and crystallographic structure of the material prepared. Raman spectroscopy measurements were recorded on a WiTec alpha300 RAS+ Confocal Raman microscope with 532 nm excitation laser at 5 mW. Scanning electron microscopy (SEM) was carried out on a crossbeam 540 FEG SEM microscope from Zeiss. The crossbeam 540 FEG was coupled with energy dispersive X-ray spectroscopy which was used for the determination of elemental composition of the samples. UV-vis absorption measurements were taken on a CARY 100 BIO UV-Vis spectrophotometer. Current-voltage (I–V) measurements were conducted on a B2900 solar management unit (SMU). Illuminated I–V measurement were performed using a Model 91150V solar simulator with solar output conditions of 1,000 W/m^2^ at 25°C and AM 1.0 G reference spectral filtering, which is the air mass coefficient used universally for measuring performance of a solar cell.

## Results and Discussion

### Characterization of MoS_2_ Nanosheets

**Figure 2:**
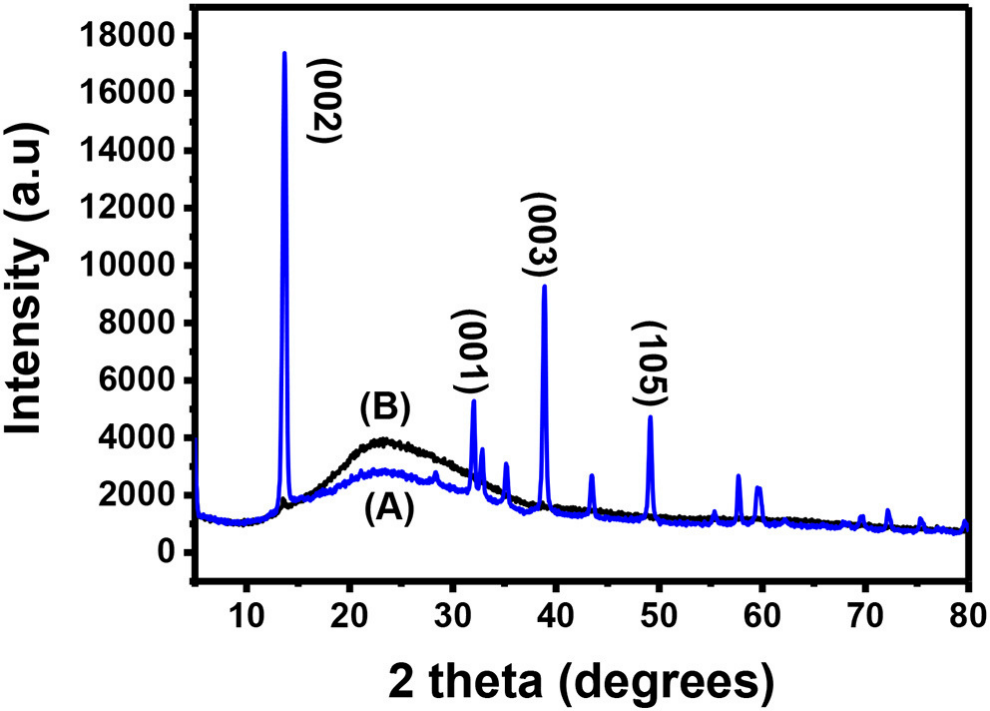
XRD spectra for **(A)** bulky and **(B)** exfoliated MoS_2_.

Bulk MoS_2_ underwent XRD characterization before exfoliation and the results were recorded. After exfoliation, the materials were spin-coated onto a microscope glass substrate, annealed and taken for XRD characterization. Figure 2A shows the XRD pattern of the bulk MoS_2_ which was indexed according to JCPDS card number 06-0097 (Štengl et al., 2014). This XRD spectrum confirms the crystal structure of MoS_2_. The XRD pattern shows a diffracted peak at 2θ = 14.4° (002) and other low intensity peaks at 2θ = 29° (004), 2θ = 33° (001), 2θ = 34° (101), 2θ = 39.56° (103), and 2θ = 49.8° (105). The peaks (002), (100), (103), and (105) are layer dependent and they can be used as an important tool to detect exfoliation. Complete disappearance of the reflections (100), (103), and (105) illustrates that exfoliation has taken place. A reduction in intensity of the (002) is also important (Ravula et al., 2015) as it indicates exfoliation.

After exfoliation, the sample was characterized and the resulting XRD pattern is shown in Figure 2B. This XRD diffractogram did not have all the peaks exhibited by the diffractogram of the bulky material. Only one peak was maintained. This peak is at 2θ = 14.3° and is indexed as the (002) peak. A direct evidence of exfoliation is the disappearance of the (100), (103), and (105) which was the case in this study. This maintained peak has a lower intensity when compared to the (002) peak for the bulky material and this was also reported elsewhere (Ma et al., 2018). The (002) peak provides useful information for determining if the MoS_2_ is bulky or if it is mono- or few-layered. Maintaining the peak with other disappearing shows that exfoliation has taken place (Park et al., 2017). A decrease in intensity of the peak suggests that exfoliation has indeed taken place and single layers are dominating the sample (Sun et al., 2014). The two XRD spectra have the (002) peak with the exfoliated MoS_2_ spectrum having a lower intensity. The difference observed between the spectra is the presence of additional peaks in the bulk MoS_2_ spectrum while the exfoliated material has only the (002) peak. As such, the difference between the two XRD spectra is due to the difference in the number of layers in the two materials.

The interlayer d_(002)_ spacing is calculated according to the (002) peak (2θ = 14.3°).

$$\begin{array}{l} \lambda =2dsin\theta \\ d=\lambda /\left(2sin\theta \right)\\ d=\frac{0.154}{2sin7.15}\\ d=0.62\;nm \end{array}$$

The interlayer d_(002)_ for the exfoliated MoS_2_ was calculated to be 0.62 nm. MoS_2_ single layer has its elements bonded in this manner: S-Mo-S. Each single-layer of MoS_2_ consists of two hexagonal planes of S atoms and an intercalated hexagonal plane of Mo atoms bound with the S atoms in a trigonal prismatic arrangement (D_3h_-MoS_6_). The symmetry space group of bulk MoS_2_ is *P*3*m*1, which is the point group D_6h_. The space group of the single layer is *P*6*m*2 which is the point group D_3h_. As a result, systems with even number of layers belong to the space group *P*3*m*1, and systems with odd number of layers to the *P*6*m*2 space group (Molina-Sanchez and Wirt, 2011; Štengl et al., 2014). The peaks shown by Figure 2 closely agrees with the reports for hexagonal 2H-MoS_2_ D_3h_ with space group *P*6*m*2 (Sun et al., 2014).

The Raman fingerprint for MoS_2_ is evidenced by vibrations at ~380 and ~410 cm^−1^. These vibrations are known as the ${\text{E}}_{2\text{g}}^{1}$ and A_1g_ peak, respectively. The ${\text{E}}_{2\text{g}}^{1}$ peak gives information about the in-plane opposite vibrations of sulfur and molybdenum atoms while the A_1g_ gives information about the out-of-phase vibrations of the sulfur atoms. Raman spectroscopy can further be used to confirm if exfoliation has indeed taken place (Kaushik et al., 2018). This confirmation can be done by comparing the Raman spectra of the exfoliated and the bulk MoS_2_ material. When moving from bulk to few layers of MoS_2_ sheets, the distance between the two Raman peaks, ${\text{E}}_{2\text{g}}^{1}$ and A_1g_, reduces indicating exfoliation has taken place.

**Figure 3:**
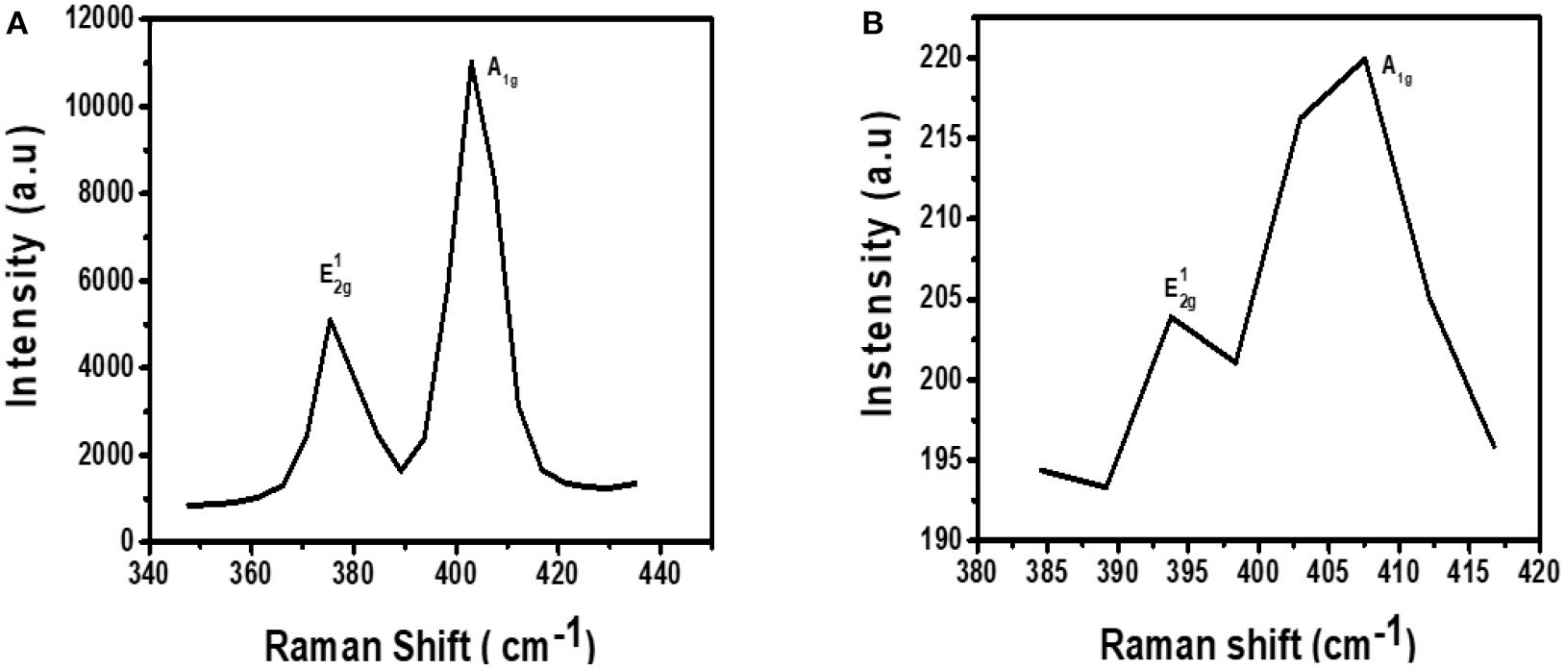
Raman spectra for **(A)** bulky MoS_2_ and **(B)** exfoliated MoS_2_.

Figure 3A shows the Raman spectrum for MoS_2_ before it was subjected to exfoliation. As expected from the Raman fingerprint of MoS_2_, the bulky material exhibited the ${\text{E}}_{2\text{g}}^{1}$ and A_1g_ peaks at 378 and 408 cm^−1^, respectively. The distance between the two peaks was found to be 30 cm^−1^.

Figure 3B shows the Raman spectrum for exfoliated MoS_2_. The spectrum contains two peaks and they are found at Raman shifts 393 and 409 cm^−1^. The distance between the two peaks was calculated to be 16 cm^−1^. This was a reduction from 30 cm^−1^ for bulky material. As such, it can be concluded that exfoliation has indeed taken place. Also, the Raman spectrum for the exfoliated material is characterized by low intensity relative to the spectrum for bulk material. This observation is similar to a previous study done by Munkhbayar et al. (2018), where they investigated the effects of exfoliation of MoS_2_ on Raman peak intensity. They observed that the intensity increases with the number of exfoliated layers suggesting a reduced intensity upon exfoliation.

**Figure 4:**
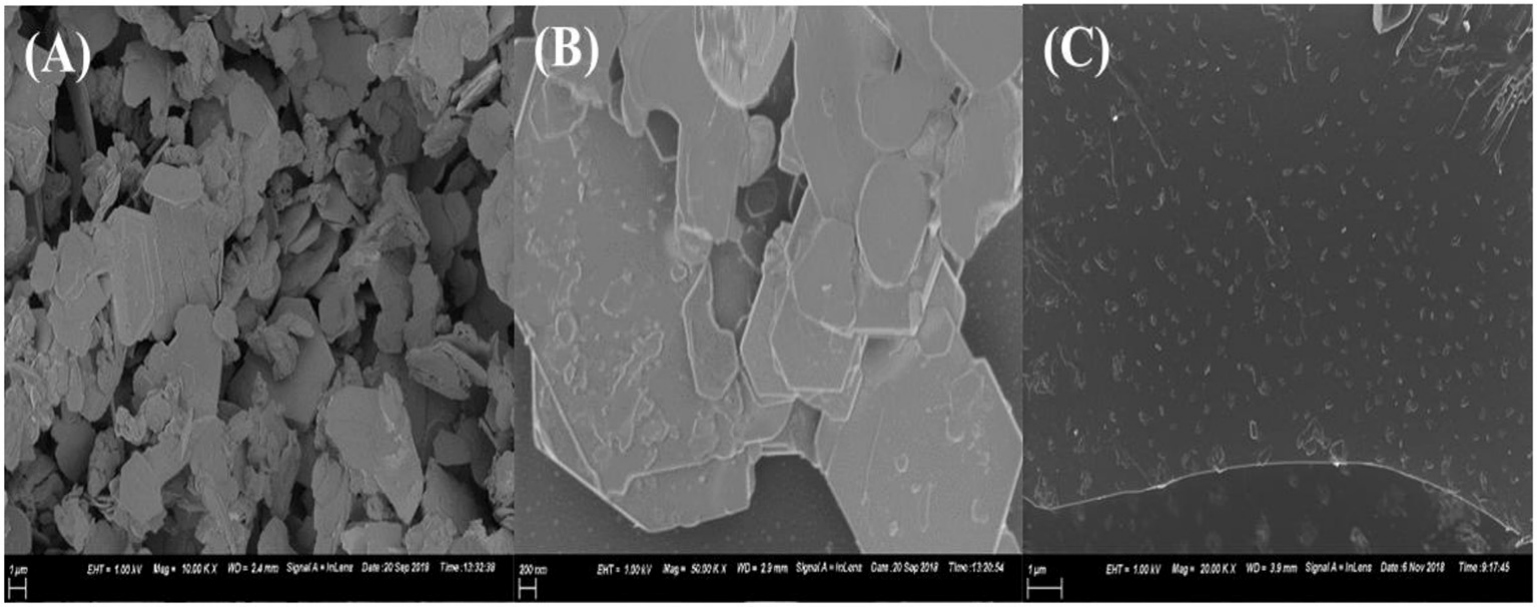
SEM micrograph of **(A)** bulky MoS_2_, **(B)** MoS_2_ exfoliated for 3 h, **(C)** MoS_2_ exfoliated for 6 h.

MoS_2_ was taken for morphology characterization using scanning electron microscopy (SEM). The aim was to confirm if indeed going from bulky to exfoliated TMDs, the formation of few-layered nanosheets can be observed. Figure 4A shows the SEM micrograph for bulky MoS_2_. The morphology observed is that of nanoflakes. Isolating each nanoflake, it can be observed that they are made up of several layers stacked together. These layers are held together by weak van der Waals forces which are easily overcome by exfoliation. Thereafter, the bulky MoS_2_ underwent exfoliation in NMP for 3 h and the sample was taken for SEM characterization. Figure 4B shows the SEM micrograph for the resulting material at a higher magnification. The morphology observed was nanosheets. However, the nanosheets were still stacked together into a few layers of different size and the layers were fewer as compared to the micrograph of the bulky material. This suggest that exfoliation has occurred. However, more time was needed for the nanosheets to be separated. The material was exfoliated further for a total of 6 h. Figure 4C shows the micrograph for the resulting material. The micrograph shows a monolayer nanosheet morphology indicating that exfoliation has taken place completely and single-layered materials were dominating the sample.

### Characterization of CuPc

**Figure 5:**
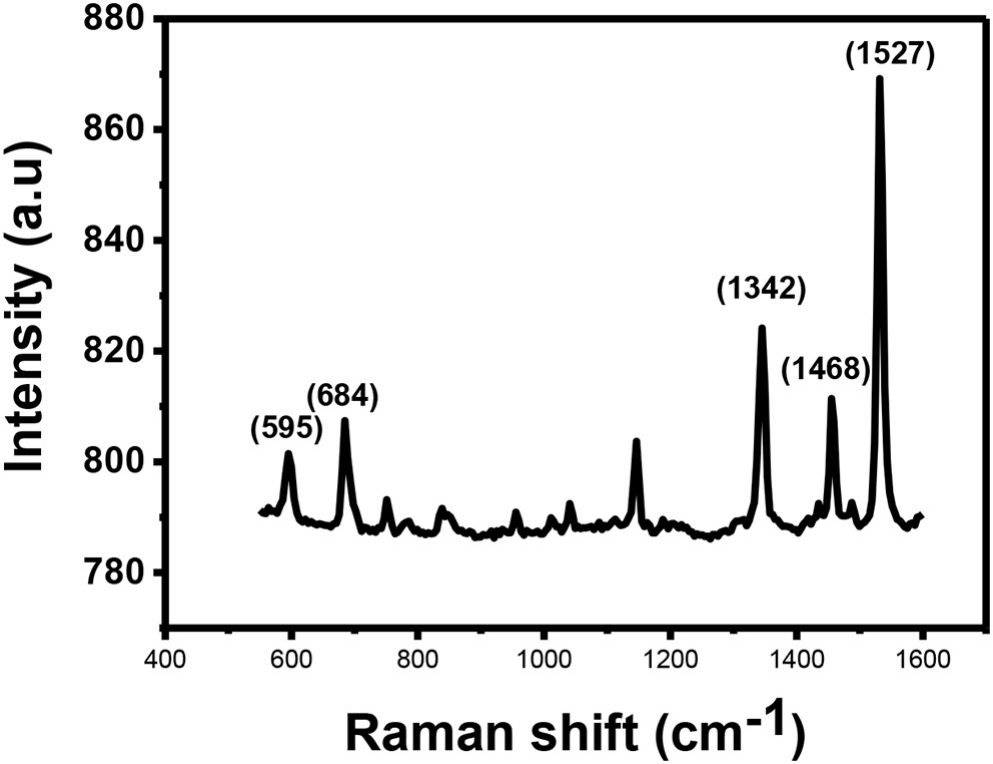
Raman spectrum for CuPc.

Figure 5 shows Raman spectra for the CuPc. The spectrum show that CuPc has a peak observed at 595 cm^−1^ known as the A_1g_ peak. This peak is related to the deformation of the benzene rings. The second peak observed is found at ~684 cm^−1^. This peak is called the B_1g_ and it is associated with benzene breathing. There are other several peaks with lower intensities found between 800 and 1,200 cm^−1^ which provide information about C-N stretching, isoindole in-plane bending, C-H bending and out of plane C-H bending (Kumar et al., 2017).

The peaks found between 1,300 and 1,600 cm^−1^ are useful for the determination of the central metal ion. Within this range, there is a coupling of two Raman spectra. The two coupled Raman spectra are B_1g_ + E_g_ and A_1g_ + B_2g_. The peaks observed in this range are located at the Raman shifts of ~1,342, ~1,468 and anywhere between 1,500 and 1,600 cm^−1^ depending on the central metal atom. The peak that is located at ~1,342 cm^−1^ provides information about pyrrole stretching. This peak is called the B_2g_ peak and it gives information about stretching of bonds, such as Cα-Nβ, Nα-Cα-Cβ, and C–C–H. The peak observed at Raman shift of ~1,468 cm^−1^ provides information about the isoindole ring stretching while the peak observed at Raman shifts between 1,500 and 1,600 cm^−1^ results due to clustering of B_1g_ and A_1g_ together into one vibration. Accurate assignment of these vibrations is important as both the vibrations are sensitive to the size of the metal ion at the center of the phthalocyanine. The peak gives information about the vibrations observed on the Cα-Nβ-Cα bridge bond. B_1g_ peak also gives information about vibrations that occur as a result of benzene stretching. The B_1g_ + A_1g_ peak for CuPc is observed at 1,527 cm^−1^.

**Figure 6:**
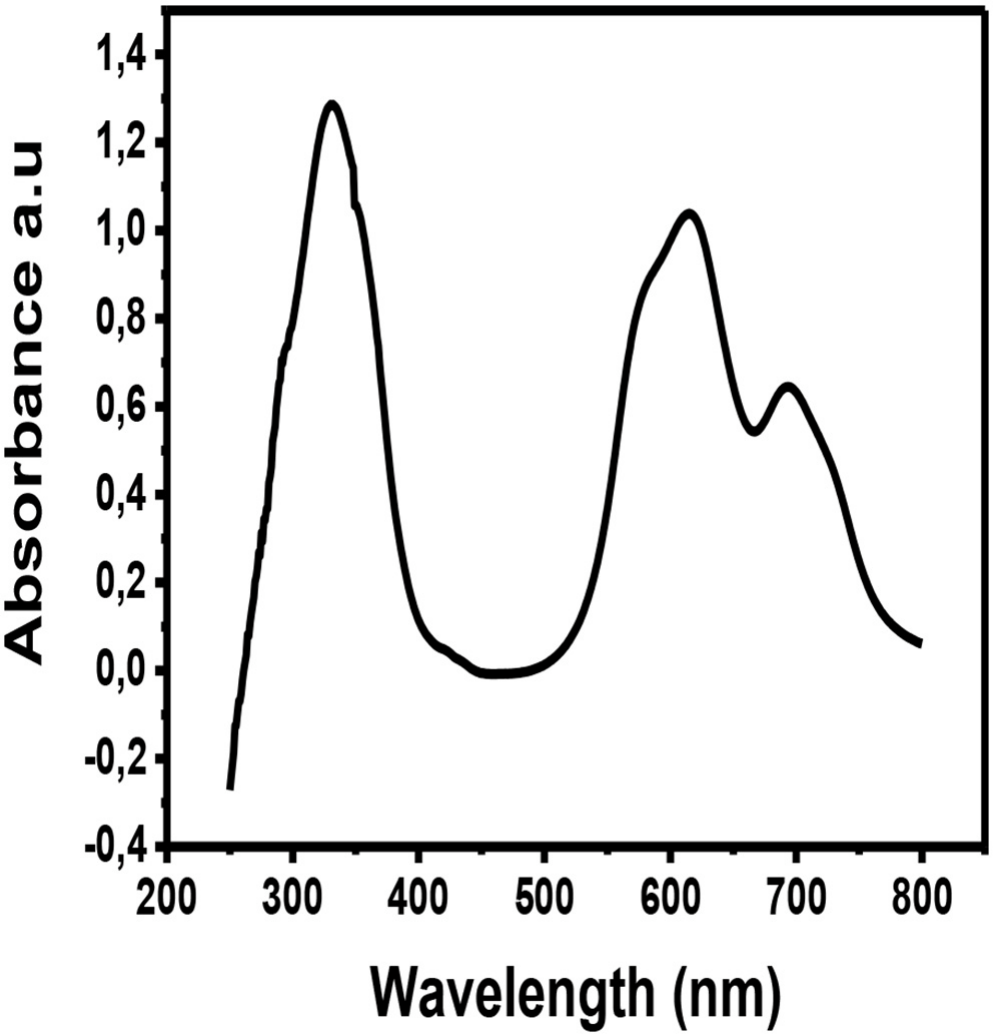
UV-Vis spectrum of CuPc.

The metallophthalocyanine was deposited by physical vapor deposition on a glass substrate and the thin film was taken for UV-Vis characterization. Figure 6 illustrates the UV-Vis spectrum of CuPc thin film. The UV-Vis spectra of CuPc exhibits three prominent absorption peaks. These absorption peaks are at wavelengths ~350, ~620, and ~700 nm. The absorption peak that occurs at the wavelength ~350 nm is associated with the Soret band of the phthalocyanine ring. This Soret band is associated with π to π^*^ electronic transitions (b_2u_ to e_g_) (Zou et al., 2018). While, on the other hand, the absorption peaks observed at ~620 and ~700 nm are associated with the Q band of the dimer and the monomer of the CuPc molecule. The Q band occurs as a result of electronic excitation of the *π* electrons from the HOMO to the LUMO of the Pc ring (a_1_ to e_g_) (Zou et al., 2018). From the UV-Vis spectrum of CuPc, the Q band fall within the range of visible light (Xu et al., 2010; Güzel et al., 2015). This suggests that this material can absorb visible light from the sun making them viable candidates for being used as photoactive layers in solar cells.

### Characterization of the CuPc/TMD Junction

**Figure 7:**
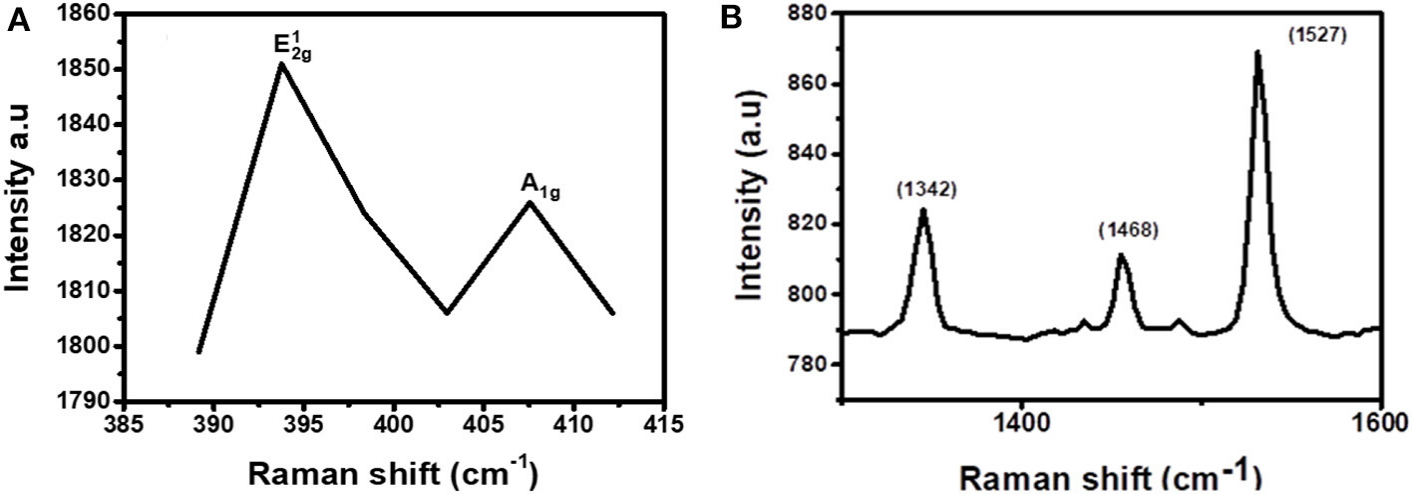
Raman spectrum for CuPc/MoS_2_ bilayer separated into two regions, **(A)** 385–415 cm^−1^ (exfoliated MoS_2_ region) and **(B)** 1,250–1,650 cm^−1^ (CuPc region).

To confirm that the layers in the junction are not disturbed and new bonds were not formed, Raman spectroscopy was conducted on the samples. For MoS_2_, the range of the Raman spectrum to be considered is 380–420 cm^−1^ while for CuPc the range to be considered is between 1,300 and 1,600 cm^−1^. As such, the spectrum was broken down into these ranges to visualize both the materials' vibrations individually. Figure 7A, which is the first part of the spectrum to be isolated, was the MoS_2_ range. From this range, the characteristic, ${\text{E}}_{2\text{g}}^{1}$ and A_1g_, peaks, were observed. This shows that the material has S and Mo atoms and it further imparts on the fact that these elements are bonded together in a manner that MoS_2_ atoms bond, meaning that the nanosheets were not disturbed upon coating over the CuPc film and no new bonds have been formed by either the Mo or the S atoms and the old bonds of MoS_2_ have not been broken as well. Figure 7B shows the spectrum range of CuPc. The peaks of CuPc at this range were maintained. This shows that the CuPc material after being coated with MoS_2_ remained intact and the atoms of CuPc have not formed new bonds and the old bonds of the materials have not been broken.

### Characterization of Doped MoS_2_

**Figure 8:**
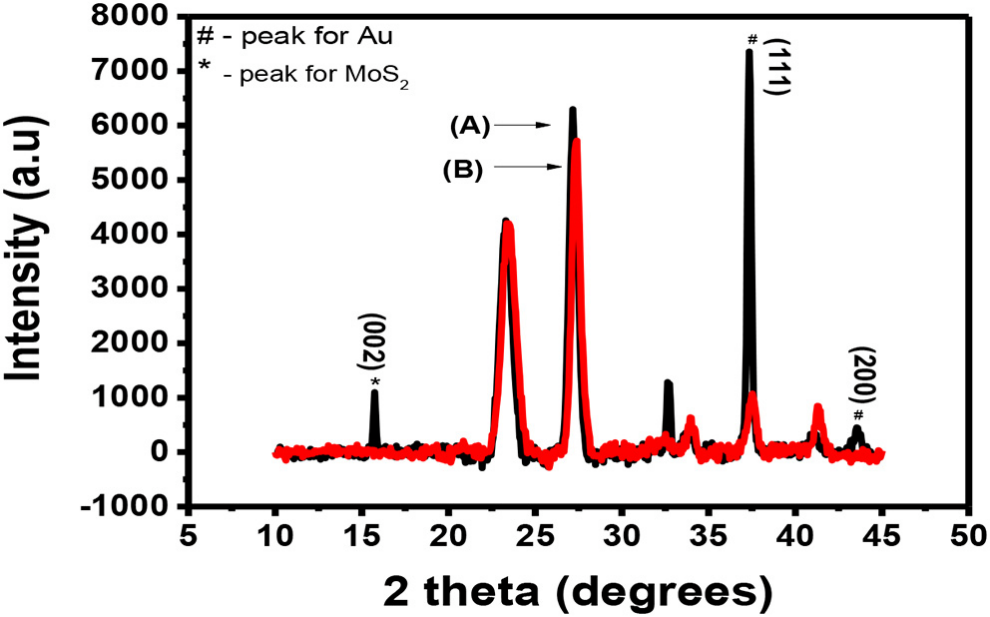
XRD spectra for **(A)** (black) Au-MoS_2_ deposited on a glass substrate and **(B)** (red) the substrate.

After doping MoS_2_ by Au, the thin film sample underwent XRD characterization. The results of these characterizations are shown in Figure 8. It is expected that the peaks of both Au and MoS_2_ nanosheets be observed on the XRD spectrum. Figure 8A shows the XRD spectra of Au-MoS_2_ with a few peaks which overlap with those of the substrate shown in Figure 8B. The weak intensity peak observed at 15.1° is indexed (002), this is a 0.8 shift from 14.3 when compared to undoped MoS_2_. This shift indicates changes in the stoichiometric composition by doping or/and a difference in the ionic radii between the main element and the dopant (Connolly, 2012). This peak occurs as a result of exfoliation of MoS_2_ into 2D nanosheets and now appears sharp. It has been found that heat treatment causes MoS_2_ to become more crystalline (Zubavichus et al., 1999; Sonto-Puente et al., 2007). The peak observed at 32.5 is indexed (100). It is not clear why this peak is observed but it has been reported previously by Qiao et al. (2017). This peak might be the (100) peak of MoS_2_ as it was also reported to be maintained after exfoliation (Niemeyer, 2015). The peaks for Au where indexed using the JCPDS. File no. 89-3697. The peaks observed at 37.8 and 44.5, indexed (111) and (200), respectively, matched with Au peaks observed from the JCPDS file no. 89-3697 (Shi et al., 2013). XRD confirmed that MoS_2_ nanosheets were successfully doped with Au.

**Figure 9:**
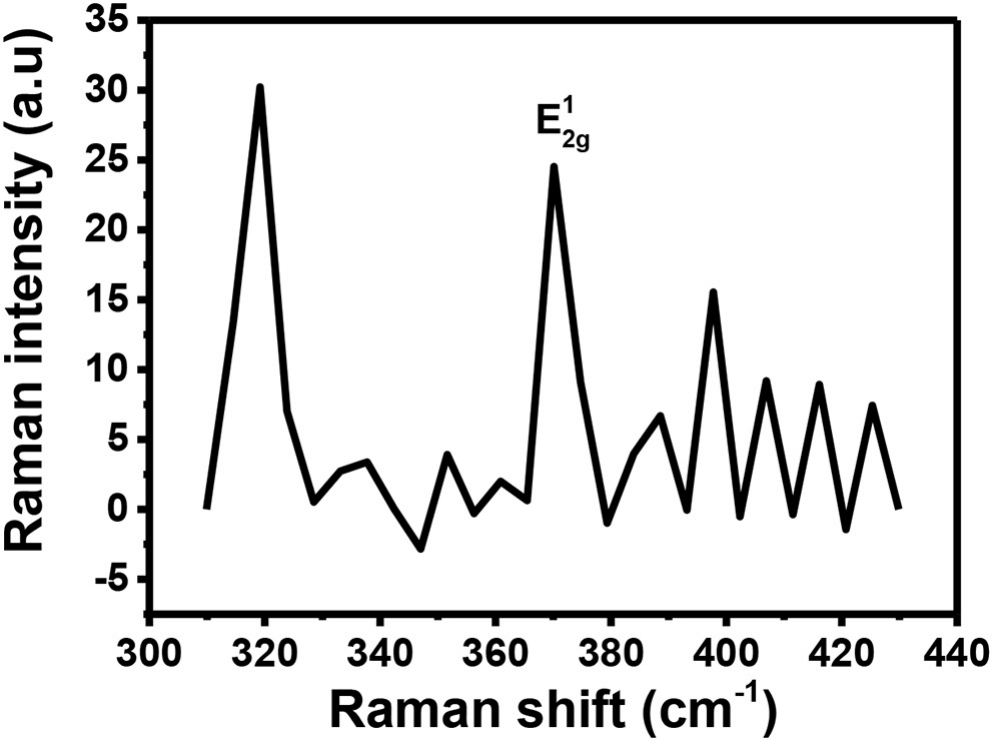
The Raman spectrum for Au-MoS_2_.

Raman spectroscopy is a powerful tool for determining if doping has successfully taken place. It can provide information about which type of doping has taken place (n or p-type doping). Generally, n-type doping of MoS_2_ results in softening of the A_1g_ peak resulting in a relative decrease in intensity (Shi et al., 2013). This type of doping is also evidenced by a reduction in the distance between ${\text{E}}_{2\text{g}}^{1}$ and A_1g_ (Shi et al., 2013). On the other hand, p-type doping generally increases the distance between the two peaks as they will move apart (Shi et al., 2013). This p-type doping also results in a Raman spectrum with an increased intensity of the A_1g_ peak (Shi et al., 2013; Singha et al., 2015). There are other cases where doping has been successfully executed on MoS_2_ but for such cases, when comparing the Raman spectra for the doped and the pristine MoS_2_, no significant change is observed (Laskar et al., 2014; Chuang et al., 2015). However, in our study, most of the expected results mentioned above were not observed upon applying p-type doping on MoS_2_ using Au. Figure 9 shows the Raman spectrum recorded for Au-MoS_2_. From this spectrum, it is observed that only the ${\text{E}}_{2\text{g}}^{1}$ peak is present. The ${\text{E}}_{2\text{g}}^{1}$ peak observed is found to be blue-shifted to 374 cm^−1^ with respect to the peak of the pristine MoS_2_. It has been reported previously that Au-S modes are observed between 250 and 325 cm^−1^ (Birte et al., 2014). From the Figure 9, there is a peak that is observed at 320 cm^−1^. The reason this peak was detected might be because of the Au-S bond in this molecule. Noisy peaks are observed at 380 cm^−1^ ≤ which may suggest that some level of crystallinity might have been lost during the doping process. The peaks were observed at relatively low intensities.

**Figure 10:**
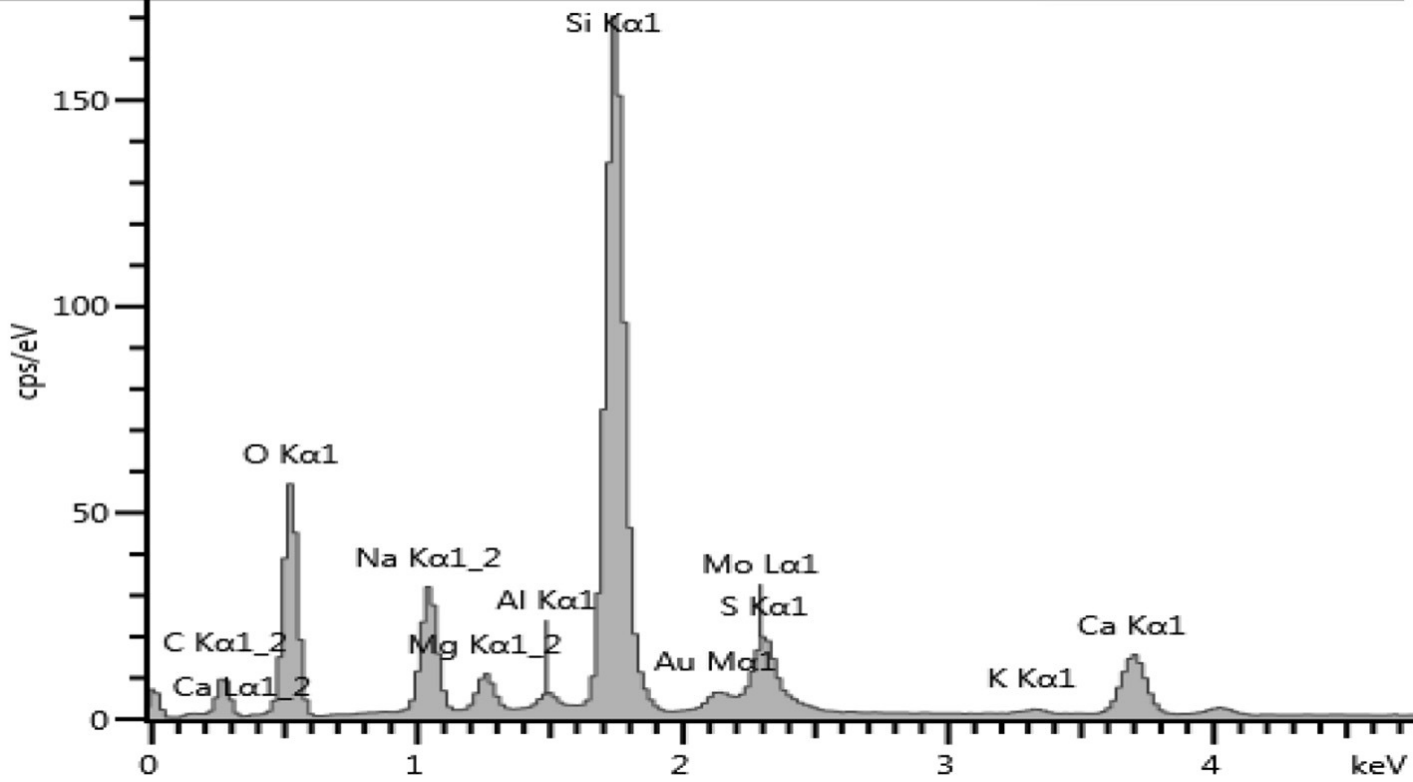
EDS spectrum of Au-MoS_2_ on a glass substrate.

After MoS_2_ underwent Au doping, the sample was taken for EDS to see the elements present in the sample. Figure 10 shows the EDS spectrum for Au-MoS_2_. The presence of Au and elements of MoS_2_ was enough to confirm that doping has occurred. This technique confirmed that all the elements making up Au-MoS_2_ are present. These elements are detected at different eV regions of the spectrum. The Au peak occurs at 2.12 eV, the Mo peaks at 2.29 and 8.40 eV, and lastly, S peak at 2.31 eV. Other elements are detected but do not form part of the sample. These are normal surface contaminants and elements of the glass substrate used.

### Characterization of Solar Cell Devices

#### Dark and Illuminated Current and Voltage (I-V) Measurements of Fabricated Solar Cell Devices

**Figure 11:**
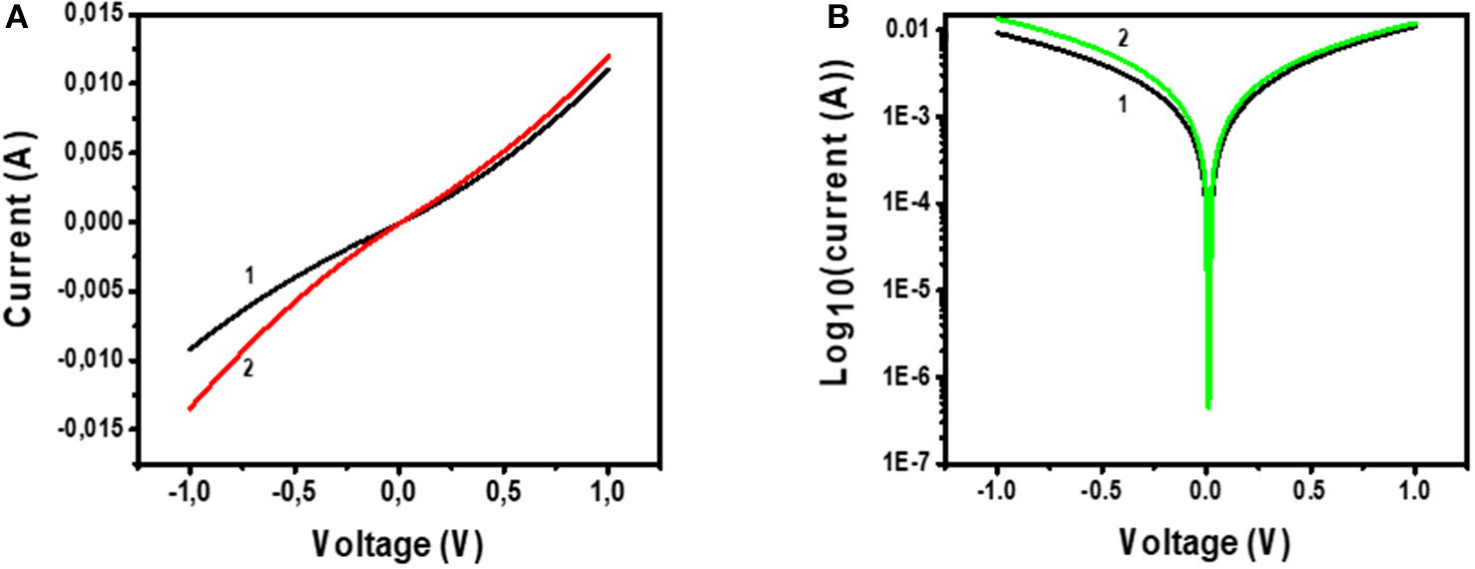
**(A)** I-V measurements of Glass/FTO/Au-MoS_2_/CuPc/MoS_2_/Au device under (1) dark conditions and (2) illuminated conditions. **(B)** Voltage against log_10_(Current) for Glass/FTO/Au-MoS_2_/CuPc/MoS_2_/Au device measured in (1) dark and (2) illuminated conditions.

Figure 11 shows the *I-V* characterization results for the device with the architecture Glass/FTO/Au-MoS_2_/CuPc/MoS_2_/Au. Figure 11A shows the *I-V* curve of the device under (1) dark and (2) illuminated conditions. Figure 11B shows a semi-log of *I-V* curve of the device under (1) dark and (2) illuminated conditions.

Considering Figure 11A, curve 1 shows that under dark conditions, when voltage is 1 V, the recorded current is 0.01 A. The shape this curve exhibits is quadratic implying that a quadratic relationship between *I-V* is observed and this type of dependence has been reported before (Fru et al., 2020). This indicates that the device is not purely ohmic and there is a slight rectification exhibited by the device. The fact that this device is slightly rectifying is confirmed by the first curve (1) on Figure 11B which is a semi-log curve of *I-V* of the device under dark conditions. The curve is asymmetric as it shows that the forward biased and the reverse biased current are not the same. The forward biased current is slightly higher than the reverse-biased current. This shows that there is rectification in the device. However, the difference between the current values for forward biased and reverse biased is small suggesting that the device could only perform at a little rectification.

A plot of *I-V* under these illuminated conditions is given in curve 2 of Figure 11A. This curve shows that a voltage of 1 V, the current is ~0.015 A. This implies that more current is generated under illuminated *I-V* measurements than in dark *I-V* measurements. This is also viewed for the semi-log plot of *I-V* of the device under illuminated conditions (Figure 11B, curve 2). A conclusion drawn from this is that the device is sensitive and can absorb visible light to generate, separate, and extract free charges. The active layer of this device, CuPc, has been shown by UV-Vis studies that it has a high sensitivity to the visible region of the electromagnetic spectrum. As such, this material is a good photon absorber and suitable for use as the photoactive layer in solar cells. Charge separation occurs at the junction between the CuPc active layer and the charge transport layers. At the MoS_2_/CuPc junction, electrons are allowed to move across the junction while holes are blocked. At the Au-MoS_2_/CuPc junction, holes move across while electrons are blocked. The separated photogenerated charges flow through the external and constitute a photogenerated current.

Despite being able to generate the electron-hole pair upon photon absorption, followed by separation of the electrons and holes at the heterojunctions, and ultimately the traveling of the electrons across the external circuit, the fabricated device showed that the relationship between *I-V* is quadratic. From the quadratic curve of *I-V*, it is not possible to measure solar cell parameters, such as fill factor and PCE. However, comparing a device exhibiting an ohmic and quadratic relationship of *I-V*, the device showing the quadratic relationship is most promising to become a solar cell. The semi-log plot for *I-V* indicates that the device shows little rectification of current. This is an important property in demonstrating that the fabricated device shows great promise in being further developed for solar cell application.

From this study, it can be seen that MoS_2_ and CuPc are compatible materials that can work together in photovoltaic systems. The deposition technique for each of the two materials also allowed them to be put against each other without interfering with the chemistry of each, proving their compatibility. Illuminated *I-*V measurements verified that the fabricated device was responsive and sensitive to visible light. CuPc was able to generate the electron-hole pair and each was transported across their respected junctions to their charge carriers. The MoS_2_ and Au-MoS_2_ were able to extract electrons and holes, respectively from the CuPc upon generation. The Au-MoS_2_ was stable and compatible with the CuPc as a clear junction was observed between the two materials. The compatibility of these materials show that they can be used together in photovoltaic devices.

## Conclusions

Mono to few-layered TMDs (MoS_2_) was successfully synthesized using a liquid-assisted exfoliation method from their bulky counterparts. To achieve this step, sonication time as well as the solvent to be used for exfoliation were carefully put into consideration. Successful synthesis of MoS_2_ was confirmed by several characterization techniques including XRD, Raman spectroscopy and SEM. The interlayer spacing for the trigonal prismatic (D_3h_ point group) MoS_2_ was found to be 0.62 nm and has a space group of *P*6*m*2. The MoS_2_ nanosheets underwent p-type doping using gold *via* a wet chemical approach. The process was successfully conducted and the resulting materials was Au-MoS_2_. Successful doping of these MoS_2_ nanosheets was confirmed using XRD, Raman spectroscopy and EDS. A photovoltaic device with the architecture Glass/FTO/Au-MoS_2_/CuPc/MoS_2_/Au was successfully fabricated for the first time in this study. The device fabricated utilized Au-MoS_2_ and pristine MoS_2_ as hole transporting layer (HTL) and electron transporting layer (ETL), respectively. For photoactive layer, this device used CuPc. The use of only CuPc as the active layer and n- and p-type MoS_2_ as the (ETL) and (HTL), respectively, resulted in the fabrication of a simple and cost-effective device. The resulting device was tested for solar cell properties and performance using dark and illuminated *I-V* characterization. The device fabricated in this study underwent dark *I-V* measurements and it showed a small amount of rectification. A conclusion drawn from this is that the device was rectifying, but not enough to perform solar cell measurements, as the dark *I-V* curve showed rectification but it was too little to allow the calculation of several solar cell parameters, such as PCE and fill factor. However, the device was not completely passive as the relationship between current and voltage was not ohmic (linear) but quadratic instead. This shows that the 2D MoS_2_, Au-MoS_2_, and CuPc are promising materials for solar cell application. From illuminated *I-V* characterization, the device had its measurements (curves) not aligned with the curves for measurements taken under dark conditions. The maximum current values recorded for the devices under illuminated conditions were higher than the current values recorded under dark conditions. As such, the fabricated device was found to be sensitive to solar radiation and hence the photoactive material (CuPc) used in the device is an adequate photon absorber and can generate electron-hole pairs sunlight. The photogenerated charge carriers were successfully separated at the junctions by the respective charge-transporting layers suggesting that the MoS_2_ (ETL) and Au-MoS_2_ (HTL) are suitable for such an application as they resulted in the device's rectification of current and could be employed in making other photovoltaic devices.

## Data Availability Statement

The original contributions presented in the study are included in the article/supplementary materials, further inquiries can be directed to the corresponding author/s.

## Author Contributions

LM conducted the lab work as part of his research thesis. JF and PK assisted LM with some of the experiments and in the preparation of the manuscript. MD was the co-supervisor of LM. NN was the supervisor of LM. All authors contributed to the article and approved the submitted version.

## Conflict of Interest

The authors declare that the research was conducted in the absence of any commercial or financial relationships that could be construed as a potential conflict of interest.
